# A New Scoring System to Differentially Diagnose and Distinguish Tuberculous Meningitis and Bacterial Meningitis in South China

**DOI:** 10.3389/fneur.2022.830969

**Published:** 2022-03-30

**Authors:** An Wen, Shi-Min Liu, Wen-Feng Cao, Yong-Liang Zhou, Chao-Qun Luo, Zheng-bing Xiang, Fan Hu, Ping Zhang, Er-Ling Leng

**Affiliations:** ^1^Department of Neurology, Jiangxi Provincial People's Hospital (The First Affiliated Hospital of Nanchang Medical College), Nanchang, China; ^2^Institution of Neurology, Jiangxi Provincial People's Hospital (The First Affiliated Hospital of Nanchang Medical College), Nanchang, China; ^3^Department of Pediatrics, Jiangxi Provincial People's Hospital (The First Affiliated Hospital of Nanchang Medical College), Nanchang, China

**Keywords:** tuberculous meningitis, bacterial meningitis, diagnosis, validation, scoring system

## Abstract

**Background:**

Tuberculous meningitis (TBM) is the most serious form of extrapulmonary tuberculosis caused by *Mycobacterium tuberculosis*, and is characterized by high morbidity and mortality. Unfortunately, it is difficult to distinguish TBM from bacterial meningitis (BM) based on clinical features alone. The latest diagnostic tests and neuroimaging methods are still not available in many developing countries. This study aimed to develop a simple diagnostic algorithm based on clinical and laboratory test results as an early predictor of TBM in South China.

**Methods:**

A retrospective study was conducted to compare the clinical and laboratory characteristics of 114 patients with TBM and 47 with BM. Multivariate logistic regression analysis was performed on the characteristics of independently predicted TBM to develop a new diagnostic rule.

**Results:**

Five characteristics were predictive of a diagnosis of TBM: duration of symptoms before admission; tuberculous symptoms; white blood cell (WBC) count, total cerebrospinal fluid WBC count, and cerebrospinal fluid chloride concentration. The sensitivity and specificity of the new scoring system developed in this study were 81.6 and 93.6%, respectively.

**Conclusion:**

The new scoring system proposed in this study can help physicians empirically diagnose TBM and can be used in countries and regions with limited microbial and radiological resources.

## Introduction

Tuberculous meningitis (TBM) is the most serious form of extrapulmonary tuberculosis (TB), especially in many countries with a high burden of pulmonary TB (1). However, more than one-half of TBM patients die or experience severe central nervous system complications despite anti-TB chemotherapy (2). Prompt diagnosis and early treatment are crucial (3) because delays in diagnosis and treatment have been identified as major determinants of outcomes (4). Cerebrospinal fluid (CSF) smear, *M. tuberculosis* culture and polymerase chain reaction are the “gold standard” for detecting *M. tuberculosis* in the cerebrospinal fluid. However, the possibility of identifying acid-fast bacilli (AFB) in CSF smears is very low, and the culture cycle of *M. tuberculosis* in CSF is very long (5). Although polymerase chain reaction methods have a higher sensitivity in detecting *M. tuberculosis* DNA in CSF samples, they also have a higher false-positive rate (6). Therefore, TBM relies more on experience for diagnosis, and is based on clinical, epidemiological, laboratory, and cerebral imaging findings (7). The aim of the present retrospective study was to create a simple diagnostic algorithm based on clinical and laboratory parameters that could be used for early prediction/diagnosis of TBM in South China.

## Methods and Materials

### Patient Recruitment

All patients admitted to the Neurology ward of Jiangxi Provincial People's Hospital (The First Affiliated Hospital of Nanchang Medical College) and Infectious Diseases Hospital of Jiangxi Province with a suspected clinical diagnosis of meningitis were included in this study. These hospitals admit patients referred from the province, which has a population of ~45.2 million. Clinical and laboratory data (blood indicators, CSF tests), and radiographic imaging (chest X-ray [CXR], computed tomography [CT], and/or magnetic resonance imaging [MRI]) results from patients with suspected intracranial infection between 2004 and 2019 were collected and retrospectively analyzed. [The data used for this manuscript was part of data collected from a separate study about meningitis. Besides, this study was approved by the Ethics Committee of Jiangxi Provincial People's Hospital (The First Affiliated Hospital of Nanchang Medical College) (NO.2003004)].

### Procedures

All patients enrolled in the study underwent standard history-taking and examination. Clinical signs and symptoms, including fever, headache, nausea and vomiting, disturbance of consciousness, duration of illness, psychiatric symptoms, cranial nerve palsies, and focal neurological impairment were recorded. Blood was drawn from all patients for routine hematological and biochemical examination, which included: erythrocyte sedimentation rate (ESR); C-reactive protein (CRP), hemoglobin, anti-TB antibody; and T cell enzyme-linked immuno-spot assay. All patients were screened for HIV infection, and each underwent routine lumbar puncture. CSF was analyzed for chloride, glucose, total and differential lymphocyte counts, and total protein concentration. CSF samples were centrifuged and stained with Gram, India ink, and Ziehl-Neelsen, and cultured on blood and chocolate agar, and Lowenstein-Jensen media. Patients underwent CXR and, if clinically necessary, cranial CT or MRI was also performed. Data were extracted from patient hospital files or computer records.

### Diagnostic Criteria

Patients were categorized into two groups based on baseline investigations. TBM diagnosis was classified as “definite,” “probable,” and “possible,” based on the case definition proposed by Marais et al. (8), who described a diagnostic scoring system including four sections regarding the evaluation of clinical characteristics, CSF findings, cerebral imaging, and evidence of TB outside the central nervous system (Table 1). Definite TBM was diagnosed or considered using the following criteria: smear microscopy for AFB in CSF or MTB, which were cultured in CSF or a commercial positive MTB nucleic acid amplification test. Patients were diagnosed with probable TBM if the total score was at least 12 (when cerebral imaging was difficult to obtain, the total score decreased to at least 10), while it was compatible between patients who received a score of 6–11, and who were possible TBM (if cerebral imaging was unavailable, the score decreased to 6–9), having the lowest 2 points of CSF or cerebral imaging criteria (9) (Table 2).

**Table 1:** Laboratory and imaging features in patients of tuberculous meningitis (TBM) and bacterial meningitis (BM).

**Laboratory features**	**ESR↑**	**CRP↑**	**Hb↓**	**TB-Ab (+)**	**T-spot (+)**	**CSF Culture/stain (+)**
TBM *n* (%)	38/86 (44%)	25/53 (47.2%)	33 (28.9%)	45 (45.5%)	22/33 (66.7%)	12 (10.5%)
BM *n* (%)	11/27 (41%)	9/10 (90.0%)	8 (17.0%)	1/30 (3.33%)	1/7 (14.3%)	13 (27.7%)
**Imaging features**	**Hydrocephalus**	**Cerebral infarction**	**Meningeal reinforcement**	**Tuberculoma**	**EEG (+)**	**CXR (+)**
TBM *n* (%)	36 (31.9%)	57 (50.4%)	74 (66.1%)	12 (10.6%)	53 (75.5%)	52 (46%)
BM *n* (%)	2 (4.3%)	19 (51.3%)	12 (25.5%)	0 (0.0%)	24/36 (66.7%)	0 (0.0%)

*ESR, Esedimentation rate (Reference range: Male 0–15 mm/h, Female 0–20 mm/h)*.

*CRP, C-reaction protein (Reference range: 0–8 mg/L)*.

*Hb, Hemoglobin (Reference range: Male ≥120 g/L, Female ≥110 g/L)*.

*TB-Ab, Antituberculous antibody*.

*T-spot, T cell enzyme-linked immuno-spot assay*.

*CSF Culture/stain (+), Cerebrospinal fluid Culture positive/Cerebrospinal fluid Gram stain or Acid fast stain positive. EEG (+), Electroencephalogram anomaly*.

*CXR (+), Chest radiograph suggestive of active tuberculosis signs of tuberculosis*.

**Table 2:** The Marais criteria for the diagnosis of TBM on admission.

	**Diagnostic score**
**Clinical criteria**	**(Maximum category score** **=** **6)**
Symptom duration of more than 5 days	4
Systemic symptoms suggestive of tuberculosis (one or more of the following): weight loss (or poor weight gain in children), night sweats, or persistent cough for more than 2 weeks	2
History of recent (within past year) close contact with an individual with pulmonary tuberculosis or a positive TST or IGRA (only in children <10 years of age)	2
Focal neurological defificit (excluding cranial palsies)	1
Cranial nerve palsy	1
Altered consciousness	1
**CSF criteria**	**(Maximum category score** **=** **4)**
Clear appearance	1
Cells: 10–500 per μl	1
Lymphocytic predominance (>50%)	1
Protein concentration >1 g/L	1
CSF to plasma glucose ratio of <50% or an absolute CSF glucose concentration >2.2 mmol/L	1
**Cerebral imaging criteria**	**(Maximum category score** **=** **6)**
Hydrocephalus	1
Basal meningeal enhancement	2
Tuberculoma	2
Infarct	1
Pre-contrast basal hyperdensity	2
**Evidence of tuberculosis elsewhere**	**(Maximum category score** **=** **4)**
Chest radiograph suggestive of active tuberculosis signs of tuberculosis = 2; miliary tuberculosis = 4	2/4
CT/MRI/Ultrasound evidence for tuberculosis outside the CNS	2
AFB identifified or Mycobacterium tuberculosis cultured from another source-i.e., sputum, lymph node, gastric washing, urine, blood culture	4
Positive commercial M. tuberculosis NAAT from extra-neural specimen	4
**Exclusion of alternative diagnoses**	

*TST, Tuberculin skin test*.

*IGRA, Interferon-gamma release assay*.

*NAAT, Nucleic acid amplifification test*.

*AFB, Acid-fast bacilli*.

The diagnosis of BM was made according to the following criteria [10]: pathogenic bacteria isolated from the CSF; or clinical meningitis with all of the following: lymphocytes and neutrophils in CSF; low glucose concentration in the CSF (<50% of that in blood); sterile blood and CSF cultures; and full recovery (without anti-TB chemotherapy) 3 months after admission (these criteria were modified to full recovery at the time of discharge).

### Statistical Analysis

Data were entered into a spreadsheet (Excel, Microsoft Corporation, Redmond, WA, USA) and analyzed using SPSS version 19.0 (IBM Corporation, Armonk, NY, USA) for Windows (Microsoft Corporation, Redmond, WA, USA). The 25 clinical and laboratory parameters of those who met the diagnostic criteria for BM and TBM were compared (Table 3). Data were compared using visual (plots/histograms) and analytical (Kolmogorov–Smirnov test) methods to determine whether they were normally distributed. Variables that were normally distributed are expressed as mean ± standard deviation; those that were not normally distributed are expressed as median ± interquartile range (M ± IR) for continuous variables. The independent samples *t*-test or Mann–Whitney U-test was used to compare continuous variables between the two groups. Frequency (%) for categorical variables and qualitative data were analyzed using the chi-squared or Fischer's exact test. Differences with *p* < 0.05 were considered to be statistically significant. The odds ratio and corresponding 95% confidence interval were calculated.

**Table 3:** Univariate analysis, comparison of the clinical and laboratory characteristics in tuberculous meningitis (TBM) and bacterial meningitis (BM).

	**TBM**	**BM**			
	***n* (%); $\bar{\text{X}}\;\pm \;\text{S}$;M ±IR**	***n* (%); $\bar{\text{X}}\;\pm \;\text{S}$;M ±IR**	**OR (95%CI)**	**Statistics**	***P*** **-value**
**Clinical features**					
Male: Female (Male %)^a^	69:45 (60.5%)	32:15 (68.1%)	0.72 (0.35–1.48)	X^2^ = 0.81	0.37
Age (y)^c^	44.50 ± 29.25	42.00 ± 28.00		Z = 0.53	0.59
Headache (%)^a^	100 (87.7%)	40 (85.1%)	1.25 (0.47–3.33)	X^2^ = 0.20	0.65
Nausea and Vomiting (%)^a^	43 (37.7%)	25 (53.2%)	0.53 (0.27–1.06)	X^2^ = 3.27	0.07
Stiff-neck (%)^a^	73 (64%)	37 (78.5%)	2.01 (0.94–4.61)	X^2^ = 3.32	0.069
Altered Consciousness (%)^a^	40 (35.1%)	20 (42.6%)	1.37 (0.68–2.74)	X^2^ = 0.79	0.37
Psychiatric symptom (%)^a^	8 (17%)	21 (18.4%)	0.91 (0.37–2.23)	X^2^ = 0.044	0.83
GCS score ^c^	15 ± 2	15 ± 6		Z = 1.916	0.055
Cranial nerve palsies (%)^a^	38 (33.3%)	9 (19.1%)	0.47 (0.21–1.08)	X^2^ = 3.24	0.072
Fundus abnormality (%)^a^	17 (14.9%)	4 (8.5%)	0.53 (0.17–1.67)	X^2^ = 1.20	0.273
Tuberculous symptoms (%)^a^	85 (74.6%)	5 (10.6%)	0.04 (0.015–0.1)	X^2^ = 55.162	0.000
Focal neurological impairment (%)^a^	41 (36%)	2 (4.1%)	0.29 (0.018–0.34)	X^2^ = 17.09	0.000
Duration of illness (d)^c^	20 ± 23.5	1 ± 6.5		Z = 7.47	0.000
**Blood tests**					
Blood WCC (x10^9^/L)^c^	7.51 ± 40.75	12.30 ± 12.00		Z = 4.56	0.000
Blood % neutrophils (%)^c^	0.75 ± 0.12	0.82 ± 0.17		Z = 2.45	0.014
Serum sodium (mmo l/L)^b^	133.29 ± 7.52	137.40 ± 7.36		t = 3.13	0.002
Blood chloride	95.89 ± 6.68	102.26 ± 6.71		t = 5.34	0.000
**CSF tests**					
Clear CSF appearance (%) ^a^	84 (73.7%)	28 (60.9%)	1.80 (0.87–3.71)	X^2^ = 2.56	0.109
CSF total WCC (x10^6^/mL)^c^	186 ± 343.25	600 ± 1212.5		Z = 4.57	0.000
CSF % neutrophils (%)^c^	0.27 ± 0.50	0.65 ± 0.53		Z = 3.33	0.001
CSF % lymphocytes (%)^c^	0.60 ± 0.58	0.2 ± 0.54		Z = 3.17	0.002
CSF/blood glucose ratio ^c^	0.35 ± 0.26	0.40 ± 0.43		Z = 0.45	0.653
CSF chloride (mmo l/L)^c^	114 ± 9.50	121 ± 8		Z = 4.77	0.000
CSF protein (mg/dL)^c^	137.25 ± 77.85	132.80 ± 60.80		Z = 1.06	0.288
CSF opening pressure (mmH_2_O)^c^	200 ± 130	180 ± 110		Z = 0.79	0.425

*GCS, Glasgow coma score*.

*Focal neurological impairment: Except cranial nerve palsies*.

*OR, Odds Ratio*.

*CI, Credibility interval*.

*WCC, White-cell count*.

*CSF, Cerebrospinal Fluid*.

*The variables were investigated using visual (plots/histograms) and analytical methods (Kolmogorow-Smirnov test) to determine whether these are normally distributed. As the variables are normally distributed, data were expressed as the “Mean ± Standard deviation ($\bar{X}\pm S$)”, if the variables did not show normal distribution (p < 0.05), data were expressed as the “median ± interquartile ranges (M ± IR)” for continuous variables; The ^b^Independent Samples T-Test or ^c^Mann-Whitney U-test was used to compare continuous variables between the two groups. And frequency (%) for categorical variables, the qualitative data was analyzed by using ^a^chi-square/Fische's exact test*.

To identify features that can be used to distinguish TBM from BM, two statistical methods were used. Firstly, variables significantly associated with TBM were included in a logistic regression analysis. Validation of the diagnostic algorithm was then performed using the logistic regression method. To determine cut-off values from the data, the value chosen was that which was closest to the upper left corner of the receiver operating characteristic (ROC) curve. Logistic regression analysis was used to build a diagnostic model. The diagnostic index (DI) of each clinical variable in the model was defined by relying on rounded β-coefficients of the model. The diagnostic index derived was: 6.645 × tuberculous symptoms + 0.196 × DSBA – 0.506 × blood WBC – 0.003 × CSF total WCC – 0.203 × CSF chloride (tuberculous symptoms were coded 1 if present and 0 if absent). Secondly, the use of a classification and regression tree (CART) model. The model was built on the basis of considering all variables individually. The range of each variable was divided into two groups to obtain the optimal separation of TBM patients from BM patients.

## Results

During the 15-year period in question (2004–2019), 380 adults with meningitis were admitted to the participating hospitals, of which 219 were excluded due to insufficient data being available to establish a definitive diagnosis or to apply a DI. Ultimately, 161 patients fulfilled the diagnostic criteria for inclusion in this study: 114 with TBM and 47 with BM. A positive CSF culture for *M. tuberculosis* and/or CSF Gram stain or acid-fast stain positivity was obtained in a total of 12 patients. There were no HIV-positive patients. Forty of the 161 adults were tested using the T cell enzyme-linked immuno-spot assay (T-spot. TB): 23 were positive (22 with TBM, 1 with BM). Among patients with TBM, 45 (45.5%) had a positive anti-TB antibody (TB-Ab), 33 (28.9%) exhibited low hemoglobin, high ESR in 38 of 86 (44%), and high CRP in 25 of 53 (47.2%). Eighty-nine of the 161 adults underwent electroencephalogram testing: 77 were positive (53 with TBM, 24 with BM). CXR and cranial CT or MRI scans were performed in all patients; chest radiographs with signs suggestive of active TB were found in 52 (46%) patients with TBM. Cranial CT/MRI scans of the patients presenting with TBM revealed hydrocephalus in 36 (31.9%), cerebral infarction in 57 (50.4%), meningeal reinforcement in 74 (66.1%), and tuberculoma in 12 (10.6%).

On univariate analysis, factors with a significant difference between the two groups included TB symptoms (mainly include fatigue, afternoon low fever, loss of appetite, night sweats and emaciation: three out of five indicators were met), focal neurological impairment, duration of symptoms before admission (DSBA), blood white cell count (WBC), blood % neutrophils, serum sodium, CSF total white cell count (WCC), CSF % neutrophils, CSF % lymphocytes, and CSF chloride (Table 3). Variables that were significant in the univariate analysis were included in the logistic regression model. Thus, it was found that five variables (TB symptoms, DSBA, blood WBC, CSF total WCC, and CSF chloride) were independently correlated with a diagnosis of TBM (Table 4). There was a conversion from continuous variables to categorical variables according to the optimal separation generated in the ROC curve. Multivariate analysis was performed to construct a diagnostic rule (Table 5). It is important to note that, to facilitate the application of the algorithm in clinical practice, the regression coefficient in the logistic regression equation was properly corrected according to the clinical situation, and the corrected regression coefficient was used as the DI for each factor, and the clinical algorithm was established based on the DI.

**Table 4:** Original multivariate logistic regression analysis.

	***β-Coefficient***	**Standard error (SE)**	**Odds ratio (95% CI)**	***P*** **-value**
Tuberculous symptoms	6.645	2.266	769.027 (0.749–0.890)	0.003
DSBA	0.196	0.075	1.217 (0.821–0.938)	0.009
Blood WBC	−0.506	0.244	0.603 (0.639–0.823)	0.038
CSF total WCC	−0.003	0.002	0.997 (0.639–0.826)	0.044
CSF chloride	−0.203	0.099	0.816 (0.656–0.822)	0.041

*WBC, white blood cell; CSF, cerebrospinal fluid; WCC, white cell count*.

**Table 5:** Weighted diagnostic index (DI) scores for dichotomized clinical variables used for diagnostic rule in admission.

**Clinical variables**	**Diagnostic index (DI)**
**Tuberculous symptoms**	
1*	4
0^#^	0
**DSBA, days**	
≥11	2
<10	0
**Blood WBC, 10** ^**9**^ **/L**	
≤ 12.25	3
>12.25	0
**CSF total WCC, 10** ^**6**^ **/mL**	
≤ 435	1
>435	0
**CSF chloride, mmol/L**	
≤ 119.5	2
>119.5	0

*WBC, white blood cell; CSF, cerebrospinal fluid; WCC, white cell count*.

1*, Tuberculous symptoms presence.

*0^#^, Tuberculous symptoms absence*.

**Figure 1:**
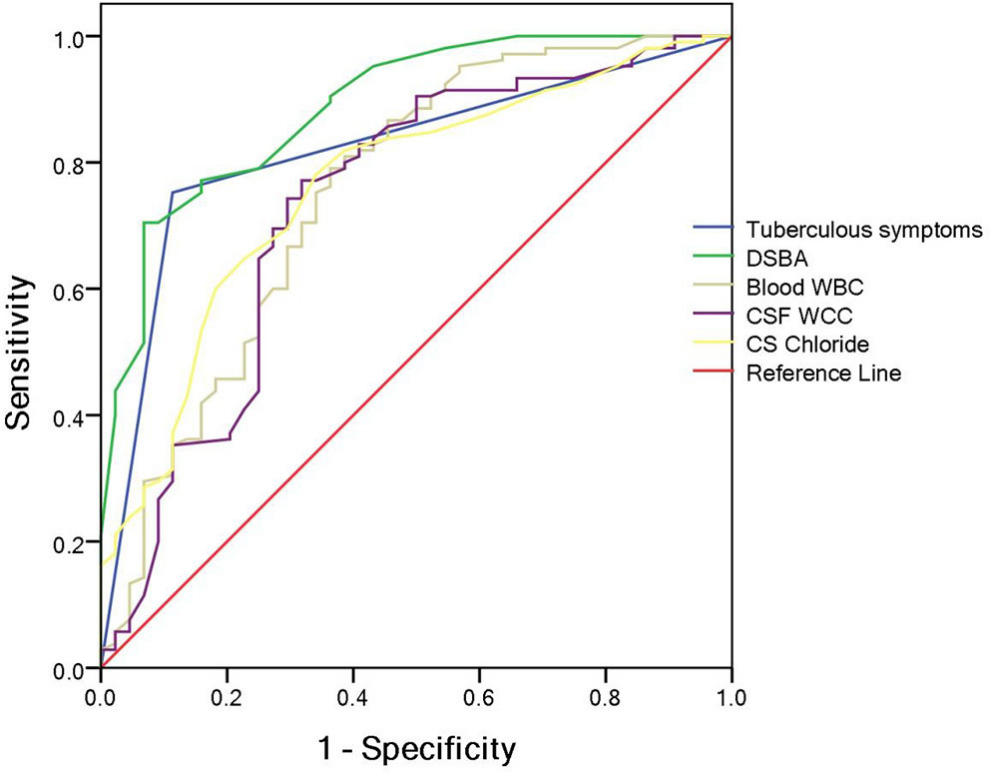
Independent predictors of tuberculous meningitis (TBM).

A classification and regression tree model was derived using the following variables: tuberculous symptoms, DSBA, blood WBC, CSF total WCC and CSF chloride. Meanwhile, the optimum cut-off value for the total diagnostic parameters to diagnose TBM patients was determined using ROC curve analysis (Figure 1): tuberculous symptoms (presence or absence), DSBA (> or < 11 days), blood WBC (> or < 12.25 × 10^9^/L), CSF total WCC (> or < 435 × 10^6^/mL), and CSF chloride (> or < 119.5 mmol/L).

**Figure 2:**
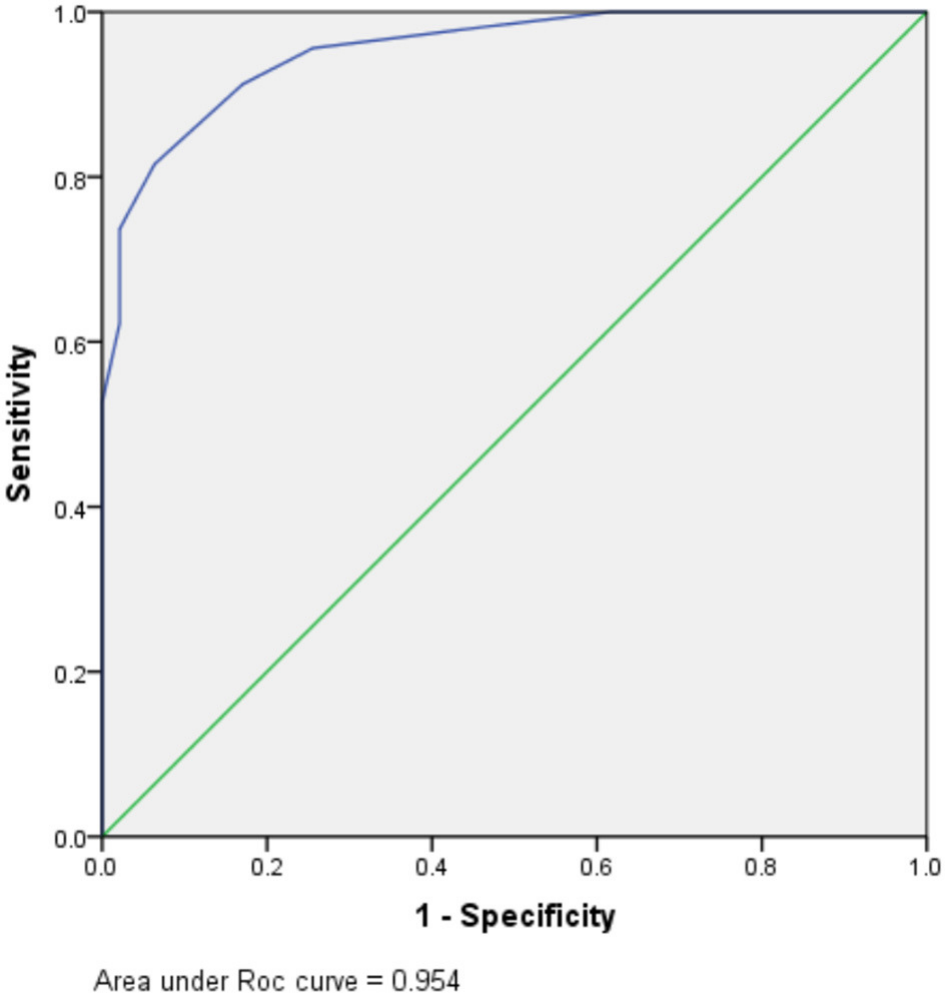
Receiver operating characteristic (ROC) for prognostic indexes from the logistic regression model.

The total DI (TDI) was calculated by aggregating all variables DIs. TDI = DI (tuberculous symptoms presence) + DI (DSBA exceeding 11 days) + DI (blood WBC count of <12.25 × 10^9^/L) + DI (CSF total WCC count of <435 × 10^6^/mL) + DI (CSF chloride of <119.5 mmol/L). The DIs for the five variables are summarized in Table 5. According to the ROC curve, the optimal segmentation point for the TDI was 7. Patients with DIs ≥ 7 were classified with TBM, while those with DIs < 7 were classified as having a version of BM. The ROC curve for the logistic model demonstrated a sensitivity of 81.6% and a specificity of 93.6%. The area under the ROC curve was 0.954 (95% confidence interval 0.924–0.983) (Figure 2).

The Marais criteria listed in Table 2 were also used to recalculate the clinical data. One hundred and fourteen patients with TBM were assessed using Marais criteria and 106 were diagnosed with TBM. Forty seven patients with bacterial meningitis were assessed using the same criteria and 32 were diagnosed with TBM. The sensitivity, specificity, positive likelihood ratio (PLR), negative likelihood ratio (NLR), positive predictive value (PPV), negative predictive value (NPV), and accuracy in this cohort were 93, 68, 2.91, 0.10, 0.88, 0.80, and 86%, respectively.

At last, another 50 patients who met the diagnostic criteria of this study were tested by this diagnostic formula:2 cases of confirmed TBM, 26 cases of clinical TBM, 10 cases of culture-confirmed bacterial meningitis, and 12 cases of clinical bacterial meningitis. The sensitivity, specificity, positive likelihood ratio (PLR), negative likelihood ratio (NLR), positive predictive value (PPV), negative predictive value (NPV), and accuracy were 96%, 86, 7.07, 0.04, 0.90, 0.95, and 92%, respectively (Table 6).

**Table 6:** Diagnostic index scores by diagnosis.

	**Marais et al. criteria**			
		**Tuberculous meningitis**	**Bacterial meningitis**	**Total**
Diagnostic index	TBM	27	3	30
	Not TBM	1	19	20
	Total	28	22	50

*Sensitivity 96%, specificity 86%, Positive likelihood ratio (PLR) 7.07, Negative likelihood ratio (NLR) 0.04,Positive predictive value (PPV) 0.90,Negative predictive value (NPV) 0.95, Accuracy 92%*.

## Discussion

Currently, TBM continues to be a very serious public problem worldwide. According to the World Health Organization Global Tuberculosis Report 2020, nearly 10 million individuals were diagnosed with TB in 2019. China alone accounts for 8.4% of new TB cases globally, and China was reported to be one of 30 TB high burden countries. In 2019, TB was the most common cause of death due to a single infectious pathogen (10). TBM remains difficult to diagnose, with high mortality and disability rates (11). Prompt diagnosis and timely initiation of appropriate therapy are crucial because delayed treatment is associated with poor outcomes (12, 13). A positive mycobacterial culture of the CSF still the “gold standard” for the diagnosis of TBM. However, a low bacterial count in the CSF leads to challenges in *M. tuberculosis* detection and diagnostic confirmation of TBM (4, 14, 15). TBM is definitely diagnosed by direct staining or culture of *M. tuberculosis* from the CSF. Nevertheless, Ziehl-Neelsen smear sensitivity can only vary between 10 and 60%, and the sensitivity can be substantially improved by meticulous microscopy of large volumes of CSF (>6 ml) (16). In addition, the sensitivity of the *M. tuberculosis* culture was within 40–60%, and the *M. tuberculosis* culture duration is 3–8 weeks, which is an unacceptable length of time for clinical decision-making (8, 17). Some studies have shown a sensitivity of ~50% and a specificity of 100% for the diagnosis of TBM using nucleic acid amplification techniques. They are a good “rule-in” test, but should never be used to “rule out” the diagnosis of TBM (18). Furthermore, a meta-analysis reported that the use of CSF interferon-gamma release assays in the diagnosis of TBM demonstrated a sensitivity of 50–70% and a specificity of 70–90%. Performance varies according to cut-off values (spots or interferon-γ concentration) and requires a substantial volume of CSF (and cell numbers) to work (19–21).

Worldwide, TBM and BM are serious life-threatening infectious diseases that require early diagnosis and treatment to reduce death and disability. Typical BM is not difficult to diagnose; however, with the widespread use and abuse of antibiotics, a large proportion of patients with BM have been treated with antibiotics in primary hospitals before admission, resulting in atypical CSF examination results and a low positive rate in bacteriology. High levels of CSF protein and low CSF glucose are commonly present in these two diseases. Meanwhile, the early clinical presentation of TBM, lack of specificity and difficult to distinguish from BM, especially partially treated BM. At the same time, clinicians are reluctant to administer patients antibacterial drugs for weeks or anti-TB drugs for months, without evidence supporting etiology, which has been a challenge for clinicians.

To facilitate clinicians in better distinguishing TBM from BM in clinical practice, some scoring systems using clinical and laboratory characteristics have been developed (22–29). In a study from Vietnam, Thwaites et al. enrolled 143 patients with TBM and 108 with BM and developed a scoring system based on the clinical and laboratory characteristics of these cases. Multivariate logistic regression analysis revealed that age, medical history, WBC count, CSF total WBC count, and CSF neutrophil ratio were independently associated with TBM. The Vietnam diagnostic rule based on the above five features demonstrated a sensitivity of 97% and a specificity of 91% (22). Validated in different populations and settings, the diagnostic rule was found to be 90% sensitive and 50–90% specific (23–27). Surprisingly, the results were not perfect when the Vietnam Diagnostic Scoring System was applied to score some with partially treated BM. A study from Turkey found that CRP level could be used as a new diagnostic parameter, and a diagnostic sensitivity of 95.5% and a specificity of 100% could be achieved when the Vietnamese rule was modified using this new index (28). Indian investigators found that the sensitivity and specificity of the new diagnostic rules after eliminating the age factor were 95.7 and 97.6%, respectively (25). In Morocco, a study by Dendane found that female sex was also an independent predictor of TBM, resulting in a sensitivity of 87% and a specificity of 96% for new diagnosis (29).

The above studies suggest that diagnostic rules behave differently in different settings and populations. For this reason, it is necessary to establish our own diagnostic scoring system to help diagnose TBM in China, and a univariate analysis of admission variables revealed a set of clinical and laboratory features with potential differentiating value (Table 1). TBM is often accompanied by tuberculous symptoms and, because TBM is a chronic disease, individuals with TBM often experience a longer DSBA, and it also has a lower blood WBC count, a lower total WCC and a lower CSF chlorine concentration. Multivariate logistic regression analysis identified six independent predictive features that distinguish TBM from BM: presence of tuberculous symptoms; DSBA > 11 days; blood WBC count (<12.25 × 10^9^/L); CSF total WCC (<435 × 10^6^/mL); and CSF chloride (<119.5 mmol/L) (Table 3).

The present study, however, had some limitations, the first of which was its retrospective design and that data for many features were unavailable. In addition, 129 patients were excluded due to incomplete data and other reasons. Second, HIV-1 antibody testing was negative for all patients included in this study. Third, the low positivity rate of CSF culture/staining makes the final diagnosis of TBM relatively weak.

The clinical features of TBM are non-specific, conventional bacteriology is widely regarded to be insensitive, and the validation of newer diagnostic methods is not complete (1, 30, 31). Therefore, accurate diagnosis and timely treatment of TBM remains challenging. Our study found that basic laboratory and simple clinical parameters can be used to help distinguish TBM from BM. The diagnostic scoring system developed by our study group demonstrated a sensitivity of 81.6% and specificity of 93.6%.

## Conclusion

The scoring system proposed in this study may help clinicians to empirically diagnose TBM, especially the high incidence of TB, and can be used in settings where microbiological diagnostic support is limited, thus ensuring rapid anti-TB treatment before other confirmation methods are available. Nevertheless, further studies are needed to validate this diagnostic scoring system.

## Data Availability Statement

The original contributions presented in the study are included in the article/supplementary material, further inquiries can be directed to the corresponding author/s.

## Ethics Statement

The study was conducted according to the guidelines of the Declaration of Helsinki and approved by the Ethics Committee at Jiangxi Provincial People's Hospital Affiliated to Nanchang University. The patients/participants provided their written informed consent to participate in this study.

## Author Contributions

AW, E-LL, and S-ML designed the study. The manuscript was written by AW, E-LL, and S-ML with the final approval. W-FC and Y-LZ did the data searches and study selection. C-QL and Z-bX did the data synthesis and created the tables and figures. All authors contributed to the article and approved the submitted version.

## Funding

This study was supported by Jiangxi Provincial Health Commission Science and Technology Foundation Grants 20204024 and 20204025.

## Conflict of Interest

The authors declare that the research was conducted in the absence of any commercial or financial relationships that could be construed as a potential conflict of interest.

## Publisher's Note

All claims expressed in this article are solely those of the authors and do not necessarily represent those of their affiliated organizations, or those of the publisher, the editors and the reviewers. Any product that may be evaluated in this article, or claim that may be made by its manufacturer, is not guaranteed or endorsed by the publisher.
